# Optical Fibre-Enabled Photoswitching for Localised Activation of an Anti-Cancer Therapeutic Drug

**DOI:** 10.3390/ijms221910844

**Published:** 2021-10-07

**Authors:** Kathryn A. Palasis, Noor A. Lokman, Bryden C. Quirk, Alaknanda Adwal, Loretta Scolaro, Weikun Huang, Carmela Ricciardelli, Martin K. Oehler, Robert A. McLaughlin, Andrew D. Abell

**Affiliations:** 1 Department of Chemistry, The University of Adelaide, Adelaide 5005, Australia; kathryn.palasis@adelaide.edu.au; 2 Institute for Photonics and Advanced Sensing, The University of Adelaide, Adelaide 5005, Australia; bryden.quirk@adelaide.edu.au (B.C.Q.); loretta.scolaro@gmail.com (L.S.); weikun.huang@adelaide.edu.au (W.H.); robert.mclaughlin@adelaide.edu.au (R.A.M.); 3 The Australian Research Council Centre of Excellence for Nanoscale Biophotonics, The University of Adelaide, Adelaide 5005, Australia; 4 Robinson Research Institute, Faculty of Health and Medical Sciences, The University of Adelaide, Adelaide 5005, Australia; noor.lokman@adelaide.edu.au (N.A.L.); alaknanda.emery@adelaide.edu.au (A.A.); carmela.ricciardelli@adelaide.edu.au (C.R.); martin.oehler@adelaide.edu.au (M.K.O.); 5 School of Biomedicine, Faculty of Health and Medical Sciences, The University of Adelaide, Adelaide 5005, Australia; 6 Centre of Research Excellence in Translating Nutritional Science to Good Health, Adelaide Medical School, The University of Adelaide, Adelaide 5005, Australia; 7 Department of Gynaecological Oncology, Royal Adelaide Hospital, Adelaide 5000, Australia; 8 Future Industries Institute, University of South Australia, Adelaide 5095, Australia

**Keywords:** biophotonics, colorectal cancer, optical fibres, photopharmacology, photoswitches

## Abstract

Local activation of an anti-cancer drug when and where needed can improve selectivity and reduce undesirable side effects. Photoswitchable drugs can be selectively switched between active and inactive states by illumination with light; however, the clinical development of these drugs has been restricted by the difficulty in delivering light deep into tissue where needed. Optical fibres have great potential for light delivery in vivo, but their use in facilitating photoswitching in anti-cancer compounds has not yet been explored. In this paper, a photoswitchable chemotherapeutic is switched using an optical fibre, and the cytotoxicity of each state is measured against HCT-116 colorectal cancer cells. The performance of optical-fibre-enabled photoswitching is characterised through its dose response. The UV–Vis spectra confirm light delivered by an optical fibre effectively enables photoswitching. The activated drug is shown to be twice as effective as the inactive drug in causing cancer cell death, characterised using an MTT assay and fluorescent microscopy. This is the first study in which a photoswitchable anti-cancer compound is switched using an optical fibre and demonstrates the feasibility of using optical fibres to activate photoswitchable drugs for potential future clinical applications.

## 1. Introduction

**Figure 1:**
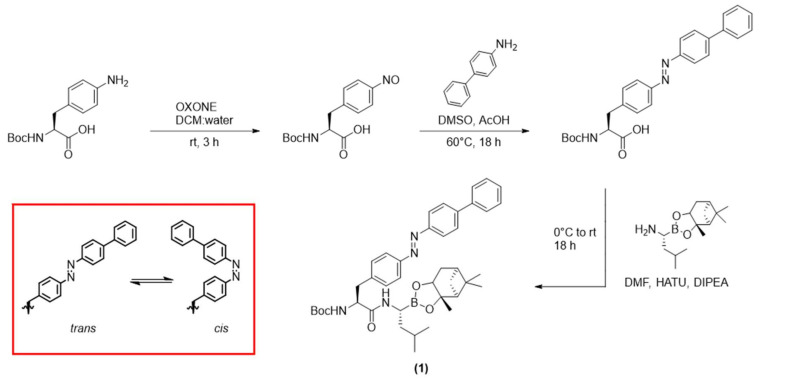
Synthesis of compound **1** (adapted from Ref. [10]) resulting in the *trans* isomer. (Inset) *trans* and *cis* isomers of azobenzene, displayed as substructures of compound **1**.

Current medical research is evolving away from conventional pharmacology towards more targeted treatments, as a means to increase selectivity and reduce side-effects [1,2]. The use of light to activate a therapeutic drug at the desired site of action, in the field known as photopharmacology, is gaining considerable attention in this regard [3]. Light has the advantage that it can be delivered with a high degree of spatiotemporal control [4,5], which enables localised, site-specific treatment [6]. Here, a functional group known as a photoswitch (e.g., an azobenzene, Figure 1, inset) is incorporated into the structure of the drug compound [7,8]. This functional group reversibly changes structure between two states upon irradiation with light of a specific wavelength. These states, referred to as *trans* and *cis* isomers for azobenzene, have distinct chemical and physical properties. While in some cases these two isomers show similar biological activity, in other cases they each give rise to a different affinity towards the biological target—these compounds are known as photoswitchable drugs, and they can enable the reversible control of bioactivity with light [9].

The photopharmacology field has advanced greatly in recent years, with photoswitchable drugs targeting ion-channels [11,12], G-protein-coupled receptors [13] and proteins [14,15,16]. They have also shown potential to treat diseases including cancers [17,18], bacterial infections [19,20] and diabetes [21]. Many of these photoswitchable drugs have shown promising results in cell culture experiments and small organisms [22,23]. However, in all cases the inability of light to penetrate tissues and reach the desired site of action is a limiting factor. In fact, there are only a few limited in vivo studies reported in mice [22,24,25], and research in the field has not yet progressed to larger animals or human clinical trials.

A critical challenge hindering the clinical development of photoswitchable drugs is the difficulty of delivering light, safely and precisely, deep inside the body to locally activate the drug where and when desired. Most photoswitchable drugs require ultraviolet light for activation, which limits penetration in turbid tissue to only a few millimetres, due to the optical scattering and absorption properties of tissue [26,27]. Several solutions have been proposed to address this limitation [22], including implantable LEDs [28], biodegradable electronics [29] and other wireless optofluidic devices [30,31]. Photoswitchable drugs that can be switched by light in the red to near infra-red region have also been investigated [20,32,33]. However, each approach suffers specific limitations. For example, implants require surgical implantation and have been observed to adversely affect animal behaviour [28].

Optical fibres have also been proposed as a promising light-delivery device to activate photoswitchable drugs [34] as their small cross-sectional area enables minimally invasive access to the body, and they can be made from a range of biocompatible materials [35,36]. Furthermore, fibre optic technology has already been demonstrated in a variety of other in vivo applications, including imaging during surgery [37], neural recording [38], fluorescent chemical sensing [39], optogenetics [40] and photodynamic therapy [41]. An advantage of optical fibres is the high efficiency with which they transport light over variable distances. Intensity of light from external light sources, such as the UV lamps or LEDs commonly used in the photoswitching literature, will vary with the inverse of the square of the distance between the light source and the compound. In contrast, optical fibres use total internal reflection within the fibre cladding to deliver light with negligible loss over distances of metres to kilometres [42]. In addition, optical fibres offer high spatial control, as the light emanates as a point source from the distal end of the fibre.

It has previously been shown that light delivered by an optical fibre can facilitate photoswitching in NMR experiments [43,44], and recent preliminary work has shown that it is feasible to use an optical fibre to activate photoswitchable drugs for diabetes treatment, pain relief and neural monitoring in vivo [45,46,47,48]. While these results are highly promising, the use of optical fibres as an effective tool to enable photoswitching of a photoswitchable drug has not yet been characterised at the molecular or cellular level, and has not been applied to the important area of photoswitchable anti-cancer agents.

Cancer is an ideal target for such studies, as many tumours begin as well-delineated and localised lesions. With improvements in cancer screening enabling earlier detection, there is a compelling clinical need for targeted treatments of these in situ tumours that avoid the debilitating side-effects of systemic chemotherapy [49]. We previously developed a photoswitchable anti-cancer drug [10] (compound **1**, Figure 1) that can selectively kill colon cancer (HCT-116) cells. In that previous work, the active photostationary state (predominantly *cis* isomer) was found to be more potent against cancer cells than the inactive (predominantly *trans*) state [10]. However, compound **1** was activated by irradiation with 352 nm light from an external UV lamp, and hence that study did not consider the need to selectively deliver light to the site of desired action. In this paper, we now show that the photoswitchable compound **1** is effectively switched by an optical fibre and its cytotoxicity is controlled in vitro, using UV–Vis absorption spectroscopy, cell viability assays and fluorescent imaging. This is the first time that switching using an optical fibre has been characterised for a photoswitchable drug, and the first report of a photoswitchable cancer therapeutic being activated by an optical fibre to selectively kill cancer cells.

## 2. Results and Discussion

Compound **1** was chosen as the photoswitchable compound for this study based on previous work, where it was shown to selectively kill colorectal cancer cells (HCT-116) after activation with 352 nm light from an external UV lamp [10]. The mechanism of cytotoxicity is through inhibition of the proteasome, and we note that compound **1** is likely to also show photoswitchable cytotoxicity against other proteasome-sensitive cancers, although the extension to other cancers is beyond the scope of this current paper.

The synthesis, photoswitching and cytotoxicity of compound **1** have been well characterised [10], making it an ideal selection for this work against the same cell line. The active state of **1** is the *cis* isomer [10]—the thermodynamically less stable state—which is an important consideration for a photoswitchable drug. This enables local activation as, once activated into the *cis* isomer at the target location, the compound will relax over time to the inactive *trans* state, which may reduce concentration of the drug in the active state throughout other parts of the body. In the case of **1**, thermal relaxation from *cis* to *trans* occurs in less than 24 h in Tris buffer [10].

**Figure 2:**
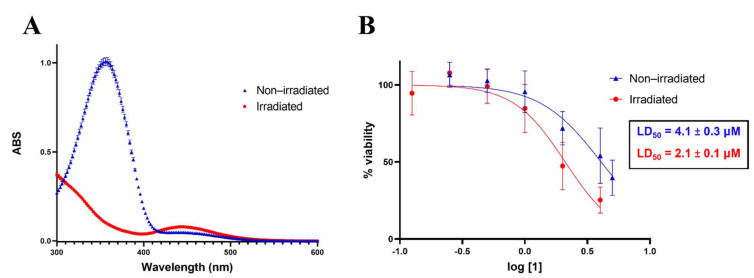
(**A**) UV–Vis absorption spectra of **1** in DMSO before and after irradiation for 10 min with 365 nm light delivered by optical fibre; (**B**) Dose–response curves (0.125–5 μM) for non-irradiated (blue) and irradiated (red) states of **1** against HCT-116 cells. An MTT assay was used to assess cell viability. The steeper curve of irradiated **1** indicates that it is more active than non-irradiated **1**. Three independent experiments, *n* = 9.

The photoswitching of compound **1** using light delivered by an optical fibre was first assessed by a comparison of the absorbance spectra of **1** in dimethyl sulfoxide before and after irradiation, as described in Section 3.4. The absorbance spectrum of the non-irradiated compound is shown as the blue line in Figure 2A. In contrast, the red line in Figure 2A shows the spectrum after 10 min irradiation with light from the 365 nm LED delivered by optical fibre. The 10 min irradiation time was chosen to ensure complete switching, based on preliminary results. There is a clear distinction between the spectra, where the absorbance at λ_max_ = 356 nm (diagnostic absorbance for *trans* azobenzene) [50] decreases, while absorbance at λ_max_ = 448 nm (diagnostic absorbance for *cis* azobenzene) [50] increases after irradiation, indicating that switching occurred [50]. Compound **1** was also successfully switched in cell media, as shown in Figure S1. These results demonstrate that the 365 nm light delivered by an optical fibre facilitates the switching of **1** from the *trans* to *cis* isomer.

Having established that light delivered through an optical fibre can switch **1** from *trans* to *cis*, it was next investigated whether the activated form of **1** could selectively kill cancer cells. HCT-116 cells were treated with varying concentrations of drug **1** and irradiated drug **1**, as specified in Section 3.6.

An MTT assay [51] was conducted to assess the cytotoxicity of **1**, where MTT salt (3-(4,5-dimethylthiazol-2-yl)-2,5-diphenyltetrazolium bromide) was added to wells containing HCT-116 cells treated with **1**. Metabolically active cells reduced the MTT salt to purple formazan crystals, which were subsequently dissolved in 0.1 N HCl in isopropanol [51]. The absorption of each well was measured at 595 nm, where a higher absorption indicated more viable cells. The absorbance of wells containing treated cells was then normalised to the wells containing control cells (no drug treatment, 0.1% DMSO), which were considered to be 100% viable.

Dose–response curves for the two states of **1** against HCT-116 cells were generated, and their respective LD_50_ values were calculated and are presented in Figure 2B. The dose–response curve of the irradiated form (red) displays a steeper curve than the non-irradiated form (blue), suggesting that the cell viability is significantly lower for those treated with the irradiated compound. The irradiated form of **1** is thus more active, with increased cell death at lower concentrations. The LD_50_ values (the concentration of a compound which is lethal to 50% of the cells) were calculated to be 2.1 ± 0.1 μM and 4.1 ± 0.3 μM for the irradiated compound and non-irradiated compound, respectively. The 2-fold difference in activity between states shows that switching was achieved by irradiation with an optical fibre, resulting in selective cell death. This is consistent with our earlier results for the irradiation of **1** with a UV lamp (352 nm), which also showed an increase in cell death for irradiated **1** compared to non-irradiated **1** [10].

Cell viability was also assessed by fluorescent staining in order to validate the result from the MTT assay. Cells treated with **1** for 48 h were stained with a combined solution of calcein acetoxymethyl ester (CAM) and propidium iodide (PI). Wells were imaged on three channels: CAM (485 nm), PI (549 nm) and brightfield.

**Figure 3:**
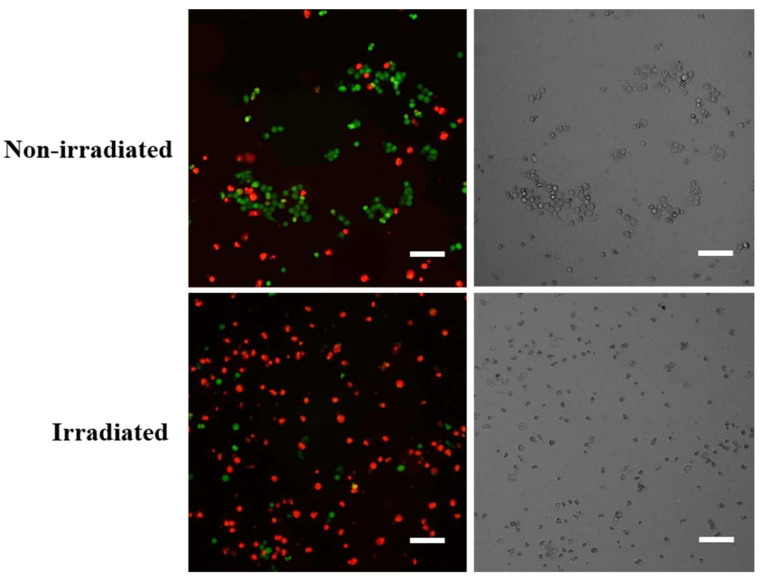
Fluorescent images of HCT-116 cells (**left**) stained with CAM (green) and PI (red) and brightfield images of HCT-116 cells (**right**) at 48 h post-treatment with 2 μM **1** in both irradiated and non-irradiated states. The CAM (green) signal indicates live cells, and the PI (red) signal indicates dead cells. A higher proportion of dead cells is shown when cells were treated with the irradiated form of **1**, suggesting irradiated **1** is more active. Scale bars, 160 μm.

Figure 3 shows fluorescence microscopy images taken of cells treated with 2 μM **1** in both irradiated and non-irradiated states. The CAM stain (green signal) indicates live cells, and the PI stain (red signal) indicates dead cells. It is clear that the proportion of dead cells is significantly higher after treatment with the irradiated form of **1** (Figure 3, bottom left), compared to those treated with the non-irradiated form of **1** (Figure 3, top left). This supports our earlier MTT assay data in demonstrating that targeted cell death was achieved by locally activating photoswitchable drug **1** using light delivered by an optical fibre.

Extending from this, preliminary studies on the direct irradiation of the cells showed that in a control experiment where HCT-116 cells were irradiated with 365 nm light delivered by an optical fibre, in the absence of **1**, no cell death was observed (Figure S2).

## 3. Materials and Methods

### 3.1. Photoswitchable Compound

The photoswitchable anti-cancer compound **1** was chosen based on previous work [10], reported there as compound **4c**, where it was shown to selectively kill HCT-116 cells after activation with 352 nm light from a UV lamp. Compound **1** was synthesised as previously reported [10], which is summarised in Figure 1.

### 3.2. Optical Fibre Setup

The photoswitchable drug **1** was irradiated with a 365 nm light-emitting diode (LED, M365FP1, Thorlabs Inc., Newton, NJ, USA) coupled with a Ø400 μm core multimode optical fibre (FT400UMT, Thorlabs Inc., Newton, NJ, USA). The fibre was attached to a custom-built mount to ensure the fibre was securely aligned directly above and orientated towards a 96-well plate containing **1**, as shown in the supplementary information in Figure S3. The fibre tip was positioned 5 mm above the top of the plate, such that the light irradiated a single well at a time. The volume of **1** that was irradiated ranged from 20 μL to 100 μL.

### 3.3. Cell Culture

The human cell line HCT-116 (colorectal cancer) [52] was chosen to allow a direct comparison with our previous work with **1** [10]. These cells were provided by the Centre for Drug Discovery and Development at the University of South Australia. HCT-116 cells were cultured in Dulbecco’s Modified Eagle Medium (DMEM, Life Technologies Australia Pty Ltd., Mulgrave, Australia), supplemented with 10% foetal bovine serum (FBS, Scientifix Pty Ltd., Clayton, Australia) and antibiotics (100 U penicillin G, 100 μg/mL streptomycin sulphate and 0.25 μg/mL amphotericin B, Sigma Aldrich Pty Ltd, Bayswater, Australia) and maintained at 37 °C in an environment of 5% CO_2_.

### 3.4. Photoswitching of **1** in Dimethyl Sulfoxide

The photoswitchable drug **1** was dissolved in dimethyl sulfoxide (DMSO, Sigma Aldrich Pty Ltd, Bayswater, Australia) at 200 μM. As the *trans* and *cis* isomers have different absorption spectra, a UV–Vis absorption spectrum was collected before and after irradiation of **1** for 10 min, with 365 nm light from the LED delivered by the optical fibre. This absorption spectrum was used to demonstrate successful switching of the compound from the inactive to active state after irradiation, via the optical fibre. The experiment was performed in triplicate and the absorption was measured on a Synergy H4 Hybrid Multi-Mode Microplate Reader (BioTek Instruments, Winooski, VT, USA).

### 3.5. In Vitro Irradiation and Dosing

The HCT-116 cells were seeded in a 96-well plate (Corning, Sigma Aldrich Pty Ltd., Bayswater, Australia) at 10,000 cells/well in growth media and incubated for 24 h at 37 °C. A 5 mM stock solution of drug compound **1** was made up in DMSO. This was diluted into phenol red-free DMEM (Life Technologies Australia Pty Ltd., Mulgrave, Australia supplemented with 1% GlutaMAX and 10% FBS and antibiotics as described in Section 3.3), to give drug solutions at final concentrations of 0.25, 0.5, 1, 2, 4 and 5 μM, at 0.1% DMSO. The growth media was removed, and drug solutions were added to the cells and left to incubate for 1 h at 37 °C. A second stock solution of **1** was made up in DMSO at 4 mM and irradiated using the 365 nm LED/optical fibre setup for 1 h. In the dark, this stock solution was diluted in phenol red-free DMEM to final concentrations of 0.125, 0.25, 0.5, 1, 2 and 4 μM at 0.1% DMSO; these drug solutions were added to the cells in the dark, and the plate was left to incubate in the dark for 1 h at 37 °C. Irradiated **1** was diluted to lower concentrations than the non-irradiated sample, as it was found to be more active in previous work [10]. After 1 h, the drugs in both plates were removed and replaced with growth media, and the plates were left to incubate for 48 h at 37 °C. This ensured that only drugs taken up by the cells could cause apoptosis, which required a 48 h incubation to be observed. The viability assay and fluorescent staining were then performed.

### 3.6. Cell Viability Assay

The cells were analysed post-treatment with compound **1** using an MTT assay [51] to determine cell viability. The growth media was removed and replaced with 100 μL per well of 3-(4,5-dimethylthiazol-2-yl)-2,5-diphenyltetrazolium bromide (MTT) (5 mg/mL 1:10 growth media, Sigma-Aldrich Pty Ltd, Bayswater, Australia) which marks viable cells. The cells were incubated for 4.5 h at 37 °C. The MTT reagent was removed and replaced with 0.1 N HCl in isopropanol (100 μL) for 10 min. Absorbance was measured at 595 nm using a Triad series multimode detector plate reader (Dynex Technologies, Inc., Chantilly, VA, USA). The inhibitory concentration (LD_50_) values were calculated from three independent experiments performed in triplicate, using curve fitting on GraphPad Prism (GraphPad Prism, version 9, GraphPad Software, Inc., San Diego, CA, USA).

### 3.7. Fluorescent Staining

Cells were also analysed 48 h post-treatment with compound **1** by fluorescent staining using calcein acetoxymethyl ester (CAM, MW = 622.55 g/mol, Invitrogen, Thermo Fisher Scientific Inc., Waltham, MA, USA) and propidium iodide (PI, (MW = 668.39 g/mol, Sigma Aldrich Pty Ltd., Bayswater, Australia). The staining solution was prepared by diluting the stock solutions of CAM (1 mg/mL in DMSO) and PI (1 mg/mL in DMSO) into final concentrations of 2 μM and 50 μg/mL, respectively, using growth media in one tube. The staining solution was warmed to 37 °C, and 150 μL of the solution was added to each well. The cells were incubated for 30 min at 37 °C, and then the staining solution was replaced with 50 μL of fresh growth media. The cells were imaged at 10× objective on a ThermoFisher Arrayscan XTI Infinity microscope (Thermo Fisher Scientific Inc., Waltham, MA, USA) on 3 channels: CAM (excitation at 485 nm), PI (549 nm) and brightfield.

## 4. Conclusions

The results presented here show that optical fibres are an effective tool in delivering light to switch an anti-cancer compound in solution and control its cytotoxicity in vitro. The field of photopharmacology has been severely hindered by a lack of such tools to enable site-specific photoswitching that is suitable for in vivo applications. We believe optical fibres are a robust and efficient light-delivery tool capable of being used to activate a wide range of photoswitchable drugs.

The UV–Vis absorption spectra of photoswitchable anti-cancer compound **1** show it is successfully switched in solution by irradiation with light delivered by an optical fibre. In vitro assays against HCT-116 colorectal cancer cells show irradiated **1** is twice as active as the non-irradiated form, with LD_50_ vales of 2.1 ± 0.1 μM and 4.1 ± 0.3 μM, respectively, as determined using an MTT assay. In support, fluorescent images of cells treated with the 2 μM drug in each state are presented, where staining with CAM and PI show more dead cells in the cells treated with activated **1**.

Our group has previously reported the development of a device incorporating optical fibres into a hypodermic needle [53]. Combined with the results presented here, this raises the potential of a device capable of delivering both a photoswitchable drug and light deep within solid tumours, such as breast, lung or liver tumours. For other cancers, such as those present in the colon or oesophagus, endoscopic fibre probes [54] are well suited to delivering light, which could be used for the localised activation of a photoswitchable drug. This approach of a targeted treatment of in situ cancers has the potential to reduce systemic effects of chemotherapy.

## Data Availability

The data that support the findings of this study are available from the corresponding author upon reasonable request.

## Supplementary Materials

The following are available online at https://www.mdpi.com/article/10.3390/ijms221910844/s1. Figure S1. UV–Vis absorption spectra of **1** in phenol red-free DMEM (as described in Section 3.4) with 2% DMSO, before and after irradiation for 5 min with 365 nm light delivered by optical fibre. Figure S2. Light-only irradiation of HCT-116 cells with 365 nm light delivered by optical fibre. CTL= control group, with no irradiation. All treatment groups contain 0.1% DMSO. 50% and 100% refers to power setting of LED (maximum power 15.5 mW). One-way ANOVA shows no statistical difference between groups. 2 independent experiments, *n* = 4. Figure S3. Schematic of optical fibre setup.
