# Supplementary material for: Optical Fibre-Enabled Photoswitching for Localised Activation of an Anti-Cancer Therapeutic Drug
Source: Int J Mol Sci. 2021 Oct 7;22(19):10844. doi: 10.3390/ijms221910844 (PMC8509559; doi:10.3390/ijms221910844)
Supplement: Supplementary file 1 [file ijms-22-10844-s001.zip › ijms-1398704-supplementary.pdf]

Supplementary Materials

Optical Fibre-Enabled Photoswitching for Localised Activation of an Anti-Cancer Therapeutic Drug

Kathryn A. Palasis [1,2,3], Noor A. Lokman [4], Bryden C. Quirk [2,3,5], Alaknanda Adwal [4], Loretta Scolaro [2,3,5], Weikun Huang [2,3,6], Carmela Ricciardelli [4], Martin K. Oehler [4,7,8], Robert A. McLaughlin [2,3,5] and Andrew D. Abell [1,2,3,*]

1 Department of Chemistry, The University of Adelaide, Adelaide, 5005, Australia; kathryn.palasis@adelaide.edu.au (K.A.P.); andrew.abell@adelaide.edu.au (A.D.A.)
2 Institute for Photonics and Advanced Sensing, The University of Adelaide, Adelaide, 5005, Australia; bryden.quirk@adelaide.edu.au (B.C.Q.); loretta.scolaro@gmail.com (L.S.); weikun.huang@adelaide.edu.au (W.H.); robert.mclaughlin@adelaide.edu.au (R.A.M.)
3 The Australian Research Council Centre of Excellence for Nanoscale Biophotonics, The University of Adelaide, Adelaide, 5005, Australia
4 Robinson Research Institute, Faculty of Health and Medical Sciences, The University of Adelaide, Adelaide, 5005, Australia; noor.lokman@adelaide.edu.au (N.A.L.); alaknanda.emery@adelaide.edu.au (A.A.); carmela.ricciardelli@adelaide.edu.au (C.R.); martin.oehler@adelaide.edu.au (M.K.O.)
5 School of Biomedicine, Faculty of Health and Medical Sciences, The University of Adelaide, Adelaide, 5005, Australia
6 Centre of Research Excellence in Translating Nutritional Science to Good Health, Adelaide Medical School, The University of Adelaide, Adelaide, 5005, Australia
7 Department of Gynaecological Oncology, Royal Adelaide Hospital, Adelaide, 5000, Australia
8 Future Industries Institute, University of South Australia, Adelaide, 5095 Australia
* Correspondence: andrew.abell@adelaide.edu.au

Switching in cell media

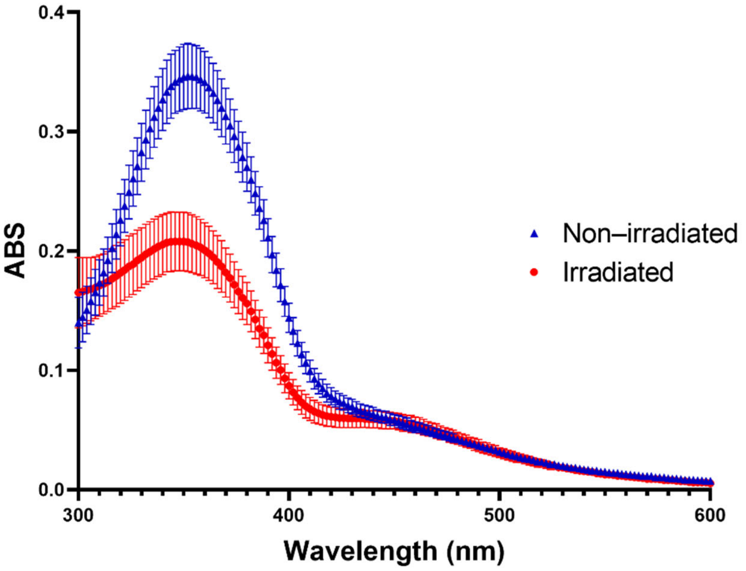

Figure S1. UV–Vis absorption spectra of **1** in phenol red–free DMEM (as described in Section 3.4) with 2% DMSO, before and after irradiation for 5 min with 365 nm light delivered by optical fibre.

Compound **1** was dissolved at 100 µM in phenol red-free DMEM (made up as described in Section 3.5) with 2% DMSO for solubility. A UV-Vis absorption spectrum was taken before (blue line in Figure S1) and after (red line in Figure S1) 5 min irradiation with light from the 365 nm LED delivered by optical fibre. Similarly to Figure 2A, there is a clear distinction between the spectra. The peak at λ_{max} = 352 nm decreases and the peak at λ_{max} = 454 nm becomes more defined, indicating successful switching from the *trans* to *cis* isomer.

Irradiation of cells in absence of 1

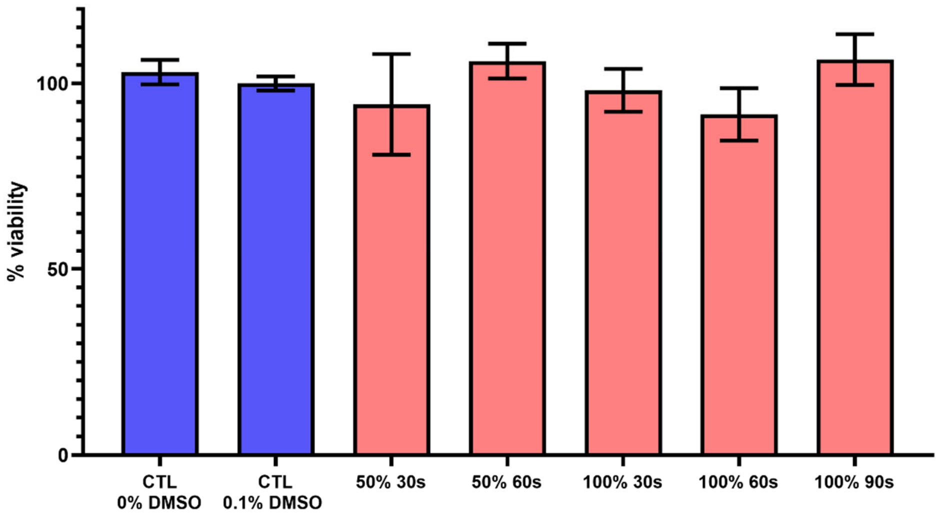

Figure S2. Light–only irradiation of HCT-116 cells with 365 nm light delivered by optical fibre. CTL= control group, with no irradiation. All treatment groups contain 0.1% DMSO. 50% and 100% refers to power setting of LED (maximum power 15.5 mW). One–way ANOVA shows no statistical difference between groups. 2 independent experiments, n = 4.

A control experiment in which HCT-116 cells were irradiated in the absence of **1** was performed and the results are presented in Figure S2. Cells were cultured as described in Section 3.3 and MTT assay used to determine viability, as described in Section 3.6. Control groups (in blue in Figure S2) were not irradiated. Treatment groups (in red in Figure S2) were irradiated with 365 nm light delivered by optical fibre, for 30 s, 60 s or 90 s and at power intensities of 50% or 100% (maximum power 15.5 mW). All treatment groups contained 0.1% DMSO, as this is the DMSO concentration used to dose cells with **1** as described in Section 3.5. A one-way ANOVA test was performed on the data, which showed no statistical difference between groups. It can therefore be concluded that irradiation with 365 nm light delivered by optical fibre does not kill HCT-116 cells alone.

Optical experimental setup

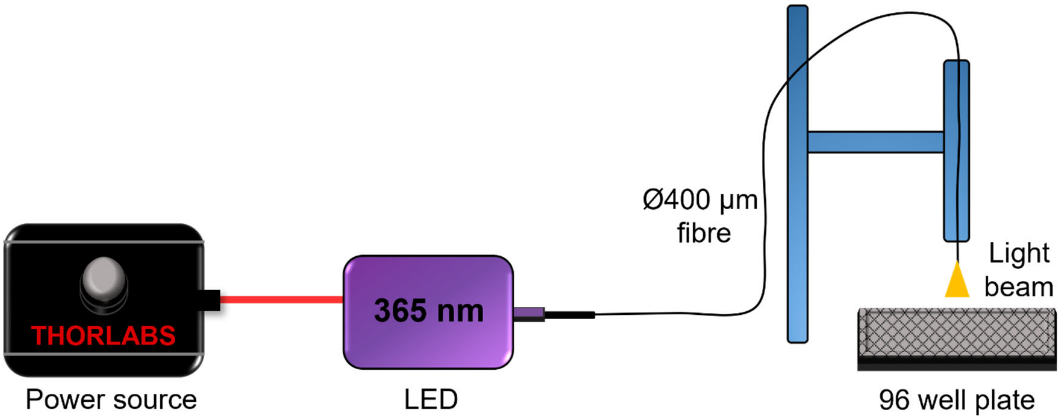

Figure S3. Schematic of optical fibre setup

Compound **1** was irradiated in a single well of a 96 well plate, using the setup shown in Figure S3**.** A 365 nm LED (M365FP1, Thorlabs Inc., Newton, NJ, USA) was coupled to a Ø400 µm core multimode optical fibre (FT400UMT, Thorlabs Inc., Newton, NJ, USA). The fibre was securely attached to a custom-built mount, and the fibre tip positioned to sit 5 mm above the top of the plate.
